# Developing an Incident Management System to Support Ebola Response — Liberia, July–August 2014

**Published:** 2014-10-17

**Authors:** Satish K. Pillai, Tolbert Nyenswah, Edward Rouse, M. Allison Arwady, Joseph D. Forrester, Jennifer C. Hunter, Almea Matanock, Patrick Ayscue, Benjamin Monroe, Ilana J. Schafer, Luis Poblano, John Neatherlin, Joel M. Montgomery, Kevin M. De Cock

**Affiliations:** 1 Division of Preparedness and Emerging Infections, National Center for Emerging and Zoonotic Infectious Diseases, CDC; 2 Ministry of Health and Social Welfare, Liberia; 3 Division of Emergency Operations, Office of Public Health Preparedness and Response, CDC; 4 Epidemic Intelligence Service, Division of Scientific Education and Professional Development, Center For Surveillance, Epidemiology, and Laboratory Services, CDC; 5 Division of Healthcare Quality Promotion, National Center for Emerging and Zoonotic Infectious Diseases, CDC; 6 Division of High Consequence Pathogens and Pathology, National Center for Emerging and Zoonotic Infectious Diseases, CDC; 7 Division of Epidemiology, Analysis, and Library Services, Center for Surveillance, Epidemiology, and Laboratory Services, CDC; 8 CDC Kenya, Center for Global Health, CDC; 9 Division of Global Health Protection, Center for Global Health, CDC; 10 Division of Global HIV/AIDS, Center for Global Health, CDC

The ongoing Ebola virus disease (Ebola) outbreak in West Africa is the largest and most sustained Ebola epidemic recorded, with 6,574 cases (1). Among the five affected countries of West Africa (Liberia, Sierra Leone, Guinea, Nigeria, and Senegal), Liberia has had the highest number cases (3,458) (1). This epidemic has severely strained the public health and health care infrastructure of Liberia, has resulted in restrictions in civil liberties, and has disrupted international travel (2). As part of the initial response, the Liberian Ministry of Health and Social Welfare (MOHSW) developed a national task force and technical expert committee to oversee the management of the Ebola-related activities. During the third week of July 2014, CDC deployed a team of epidemiologists, data management specialists, emergency management specialists, and health communicators to assist MOHSW in its response to the growing Ebola epidemic. One aspect of CDC’s response was to work with MOHSW in instituting incident management system (IMS) principles to enhance the organization of the response. This report describes MOHSW’s Ebola response structure as of mid-July, the plans made during the initial assessment of the response structure, the implementation of interventions aimed at improving the system, and plans for further development of the response structure for the Ebola epidemic in Liberia.

A clearly defined chain of command and organizational structure, effective resource management, and advanced planning are important aspects of an emergency response. An IMS is a standard structure based on these principles that is used in large and small-scale incidents throughout the United States at the federal, state, and local level (3). CDC has adapted IMS principles in managing their responses to public health emergencies, which in addition to the command, operations, logistics, planning, and finance/administrative functions, also includes scientific/public health response roles (4).

## Initial Ebola Response Structure and Efforts to Improve Response Structure

**FIGURE 1:**
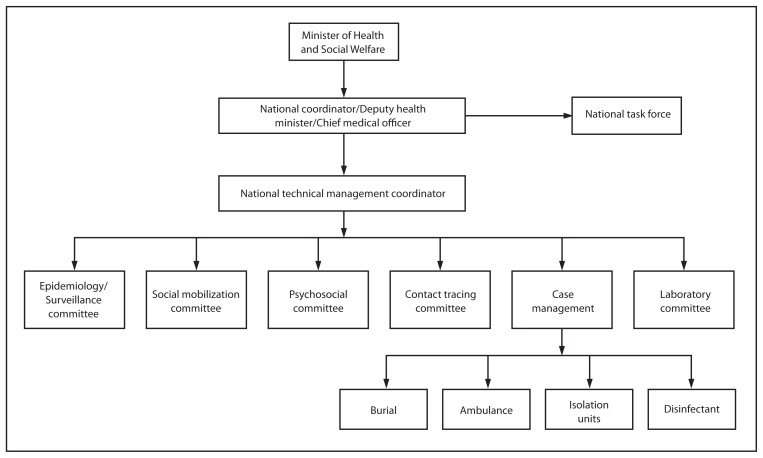
Ministry of Health and Social Welfare Ebola response framework — Liberia, July 2014

The national response system that was initially established by MOHSW employed several IMS elements. For example, a national coordinator for the Ebola response was identified. This position was held by MOHSW’s deputy health minister/chief medical officer. Additionally, daily meetings were held that were attended by the heads of each technical committee deemed important for the operational response to the epidemic: epidemiology/surveillance, social mobilization (responsible for communication of key messages), psychosocial (responsible for ensuring adequate social and mental health support for patients and families affected by Ebola infection), contact tracing, case management, and laboratory. MOHSW leadership recognized that this organizational structure (Figure 1) and the overall response could be further optimized and sought to implement improvements with technical support from CDC.

Several areas were identified where the response structure might benefit from adjustment. The initial response structure implemented by MOHSW represented what would be recognized as the scientific response section of a public health response (4). The deputy health minister was responsible for not only MOHSW’s Ebola response framework as the national coordinator but also for other, non–Ebola-related public health responsibilities as the country’s chief medical officer (e.g., overseeing the county-level delivery of health care in outpatient and inpatient settings and overseeing prevention and control programs, including those related to immunization, human immunodeficiency virus, tuberculosis, and malaria) (5). The national coordinator did not have a deputy to serve as an alternate decision-maker when the national coordinator was unavailable (e.g., when attending higher level meetings). In addition to overseeing the national response, MOHSW’s span of control over the response was stretched because it also provided direct support for many activities in the counties surrounding the capital (e.g., assisting with case management and coordinating ambulance and burial transport). Regarding meetings, each morning the national coordinator presided over a national task force meeting, during which presentations were made by technical committee heads. The meeting included numerous partner organizations working in Liberia on the Ebola response (e.g., representatives of the World Health Organization [WHO], public health agencies from other countries, and nongovernmental organizations), with attendance exceeding 50 persons. The numerous comments and input from this large group made it difficult to develop clearly articulated action items. Furthermore, when logistics challenges were identified (e.g., lack of fuel or vehicles to transport teams to investigate potential cases, or to transport a burial team), there was not a single point of contact among the large assembled group to provide the logistical and administrative support to respond to these needs.

## Improvements to the Ebola Response Structure

**FIGURE 2:**
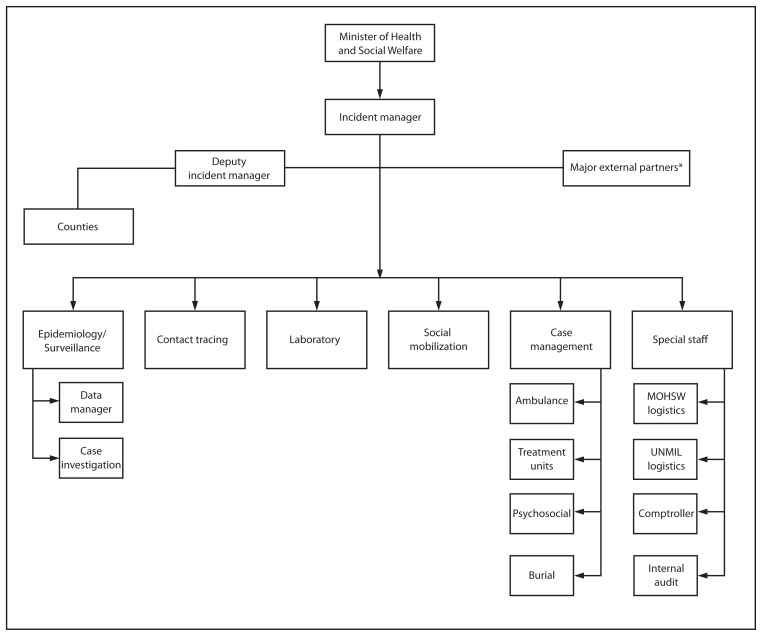
Ministry of Health and Social Welfare Ebola response incident management system — Liberia, August 2014 * Including the World Health Organization, CDC, Medécins Sans Frontières, UNICEF, and the U.S. Agency for International Development.

MOHSW developed plans to further refine the command and control structure; develop an IMS general staff section to support the scientific response section with logistical, administrative, and planning components; identify how best to link the national IMS to the county-level response and external partners; and improve the organization of IMS meetings to ensure response objectives had clearly identified action items and that these action items were acted upon. Where possible, efforts were made to work within the existing MOHSW framework to facilitate implementation of the changes (Figure 2).

Regarding command and control, on August 10, 2014, the Minister of Health and Social Welfare appointed an incident manager (IM) responsible for only the Ebola response, chairing a 9:00 am incident management meeting, and establishing, following-up, and adjusting the response priorities and objectives. This allowed the deputy health minister/chief medical officer to focus on other pressing, non–Ebola-related public health activities. In terms of organizational structure, a deputy IM, operations chief, and planning chief were identified. The deputy IM had the authority to step in and function as the IM, to ensure the response continued to have command and control when the IM was in higher level coordination meetings related to the response. The deputy IM also convened and guided a regular logistics meeting attended by MOHSW and partners with logistical interests or resources and chaired a subcommittee to address county level issues. This county-specific subcommittee served as the forum where technical, administrative, and logistical needs for the county responses could be raised. The deputy IM and all technical and general staff committees reported directly to the IM. With respect to IM meetings, each key Ebola response committee was instructed to have the chair (or an alternate with decision-making authority) attend. An agenda was implemented that focused meeting discussions on the key actions completed during the previous 24 hours, actions to be completed during the next 24 hours, and major challenges being faced. The meetings also included a representative from the logistics and finance section (responsible for keeping track of the financial resources available to MOHSW for the managing the response). These changes allowed for more regular reporting of key logistical items to the IM, such as availability of personal protective equipment and regular budget status reports. A task listing was implemented assigning responsibility and due dates for action items as they were identified, and more detailed meeting minutes were prepared and issued the same day as the meeting. The addition of logistical and financial/administrative general staff facilitated completion of the objectives identified by the IM. When expertise did not exist within MOHSW, assistance was sought from other response partners (e.g., logistics support was sought from the United Nations Mission in Liberia, given the mission is a well-resourced organization in Liberia with a track record of timely and efficient movement of personnel and equipment across the country). To facilitate the ability of MOHSW to reach out to external partners, the IMS included liaisons with key external stakeholders involved in the coordination of international partners and provision of essential supplies and technical expertise, such as WHO, CDC, Medécins Sans Frontières, UNICEF, and the U.S. Agency for International Development (Figure 2).

What is already known on this topic?

The ongoing Ebola virus disease (Ebola) outbreak in West Africa is the largest recorded outbreak in history, and the response to the outbreak involves numerous domestic and international partners. A clearly defined chain-of-command and organizational structure, effective resource management, and advanced planning are important aspects of an emergency response. An incident management system (IMS) is a standard tool based on these principles, and CDC has adapted IMS principles in managing numerous public health emergency responses.

What is added by this report?

During July and August 2014, the Liberian Ministry of Health and Social Welfare (MOHSW), in consultation with CDC, refined their response to the Ebola outbreak through the institution of an IMS. This system included the establishment of a dedicated incident manager responsible for defining the specific goals and objectives of the response; the creation of additional support staff positions to aid the logistical, administrative, and financial components of the response; and enhancement of the efficiency of incident management meetings.

What are the implications for public health practice?

IMS provides an organized response framework, which will allow MOHSW to more rapidly and effectively address the burgeoning Ebola outbreak. Additionally, the findings in this report might also be useful in other settings where IMS has not been used previously and is being considered for the first time for the management of public health emergencies.

The revised IMS structure did not replace the national task force, which consists of a higher-level interministerial coordination group and key external partners. Thus, ongoing work is need to integrate the MOHSW response structure into this overarching national Ebola response framework. Also, the current “planning horizon” is about 24 hours. Continued development of a planning section in the IMS, to look beyond this limited timeframe, is required to anticipate potential problems and develop contingency plans.

## Next Steps

The changes described represent work done during mid-July through mid-August. MOHSW colleagues, with technical assistance from CDC, will continue refining the IMS during the next 6–9 months. During this period, there are several anticipated objectives, the first of which is to ensure the IM designates all priorities for the subsequent 24–48-hour operational periods. Development of a robust planning section to look beyond this 24–48-hour timeframe also will occur. Because much of the operational component of the response (case identification and contact tracing) resides at the county level, there needs to be ongoing information exchange with the counties and MOHSW through the subcommittee chaired by the deputy IM. This information exchange will need to focus on ensuring sufficient logistical support for these county-level operations. Finally, a permanent emergency operations center at MOHSW is planned to serve as a location to receive calls and reports, to replace the current model of direct reporting of information to the scientific response section chairs and IM leadership.

## Conclusion

MOHSW has readily adopted the concept of IMS during the early months of this response to align their national response structure with well-recognized emergency management principles. Clearly, the institution of an IMS in Liberia for the management of the Ebola response will be an evolutionary process, not only because the concepts are new to MOHSW, but because these concepts are also new to the other ministries with which MOHSW coordinates and to the political structure to which MOHSW reports. It is hoped that by instituting an organized response framework, which IMS provides, MOHSW will be able to more rapidly and effectively deal with the burgeoning Ebola outbreak in Liberia. The findings in this report might also be useful in other settings where IMS has not been used previously and is being considered for the first time.

## References

[1] Incident Management System Ebola Epidemiology Team, CDC; Ministries of Health of Guinea, Sierra Leone, Liberia, Nigeria, and Senegal; Viral Special Pathogens Branch, National Center for Emerging and Zoonotic Infectious Diseases, CDC (2014). Ebola virus disease outbreak—West Africa, September 2014. MMWR.

[2] CDC (2014). Ebola in Liberia.

[3] Emergency Management Institute (2008). ICS review document.

[4] Papagiotas S, Frank M, Bruce S, Posid JM (2012). From SARS to 2009 H1N1 influenza: the evolution of a public health incident management system at CDC. Public Health Rep.

[5] Ministry of Health and Social Welfare (Liberia) (2014). Department of Health Services.

